# Cervical Cancer Prevention and High-Risk HPV Self-Sampling Awareness and Acceptability among Women Living with HIV: A Qualitative Investigation from the Patients’ and Providers’ Perspectives

**DOI:** 10.3390/curroncol29020047

**Published:** 2022-01-26

**Authors:** Daisy Le, Annie Coriolan Ciceron, Min Jeong Jeon, Laura Isabel Gonzalez, Jeanne A. Jordan, Jose Bordon, Beverly Long

**Affiliations:** 1 School of Nursing, The George Washington University, Washington, DC 20006, USA; acoriolan@gwu.edu (A.C.C.); mjjeon@gwu.edu (M.J.J.); 2 Department of Prevention and Community Health, Milken Institute School of Public Health, The George Washington University, Washington, DC 20052, USA; lgonz464@gwmail.gwu.edu; 3 District of Columbia Center for AIDS Research (DC CFAR), Washington, DC 20052, USA; jajordan@gwu.edu; 4 GW Cancer Center, The George Washington University, Washington, DC 20052, USA; 5 Department of Epidemiology, Milken Institute School of Public Health, The George Washington University, Washington, DC 20052, USA; 6 Washington Health Institute, Washington, DC 20017, USA; jbordon@dc-whi.org; 7 College of Medicine, Florida State University, Tallahassee, FL 32304, USA; beverly.long.11@gmail.com

**Keywords:** human papillomavirus, HR-HPV self-sampling, women living with HIV, cervical cancer screening, cervical cancer, cancer prevention

## Abstract

Routine cervical cancer screening is important for women living with HIV (WLH) due to the greater incidence and persistence of high-risk HPV (HR-HPV) infection. HR-HPV self-sampling has been proposed to overcome barriers to in-office cervical cancer screening in underserved populations. However, little is known about baseline knowledge of HR-HPV and the acceptability of HR-HPV self-sampling among WLH. This paper describes WLH’s experiences and needs regarding cervical cancer screening, specifically HR-HPV self-sampling, and seeks to reconcile their experiences with the views of their providers. In total, 10 providers and 39 WLH participated in semi-structured interviews and group discussions, respectively. Knowledge of cervical cancer and HR-HPV was generally limited among WLH; when present, it was often due to personal experience of or proximity to someone affected by cervical cancer. Most WLH were not familiar with HR-HPV self-sampling but, despite some of the providers’ skepticism, expressed their willingness to participate in a mail-based HR-HPV self-sampling intervention and highlighted convenience, ease of use, and affordability as facilitators to the uptake of HR-HPV self-sampling. The experiences identified can be used to guide patient-centered communication aimed at improving cervical cancer knowledge and to inform interventions, such as HR-HPV self-sampling, designed to increase cervical cancer screening among under-screened WLH.

## 1. Introduction

Women living with HIV (WLH) bear a disproportionate risk of high-grade cervical intraepithelial neoplasia (CIN2+) and invasive cervical cancer due to greater incidence and longer persistence of high-risk human papillomavirus (HR-HPV) infection in this population [1,2,3]. Persistent detection of HR-HPV genotype(s) in cervicovaginal specimens confers an increased risk of developing CIN2+, while a negative HR-HPV test predicts a less than 2% risk of developing CIN2+ within 3 years [4,5,6,7]. Additionally, low (<200) CD4 count and high viral load are associated with the development of invasive cervical cancer, further increasing this risk in WLH [8,9]. A recent analysis using longitudinal data from a cohort of women receiving HIV care in Washington, District of Columbia (DC) found a cervical cancer incidence of 0.7 per 1000 person-years, which is over ten times higher than that for the general population in the United States (U.S.) [10,11,12].

Cervical cancer is preventable through early detection with regular Papanicolaou (Pap) and/or HR-HPV screening. Current guidelines recommend initiating cervical cancer screening at HIV diagnosis but not before age 21. Cervical cytology is performed annually for three years, then every three years if normal in women age 21–29. In women age 30 or greater, follow-up cytology and HR-HPV co-testing is performed every 3 years if the initial co-testing result is negative [13]. While there is a paucity of research regarding knowledge of cervical cancer and HR-HPV screening among WLH, limited evidence indicates suboptimal knowledge of HR-HPV transmission and cervical cancer prevention [14,15,16]. This knowledge gap may lead WLH to under or overestimate their risk of cervical cancer [14,15,16] and could affect their ability to make informed and sustainable decisions related to cervical cancer prevention. Reliable risk calculation is especially important since WLH face additional barriers to screening, including social and economic factors, stigma specific to co-infection, perceived pain, embarrassment, bodily modesty, fear of diagnosis of serious illness, and limited access to female providers [14,15,16].

To improve cervical cancer screening rates, self-collection of cervicovaginal samples for HR-HPV testing has been proposed as an alternative to traditional office-based screening [17]. Primary HR-HPV screening has been FDA-approved for cervical cancer screening in women 25 years and older since 2014. Several studies have demonstrated appropriate safety, sensitivity, specificity, and acceptability of HR-HPV self-collection [17,18,19,20], particularly among underserved and vulnerable populations [21,22,23,24], e.g., similar sensitivity has been reported for the detection of CIN2+ in self-collected compared to clinician-collected samples (92–99%) [19,25]. In large international studies, up to 40% of under-screened women used and returned a HR-HPV self-test that was sent via mail [26,27]. Given this success, several countries integrated mail-based HR-HPV self-testing for under-screened women into their national screening programs [28,29]. This approach has not been adopted as the standard of care in the U.S. [22]. Since U.S. cervical cancer screening rates remain suboptimal [30], self-sampling represents an opportunity to strategically encourage HR-HPV testing among under-screened populations [31]. WLH may disproportionately benefit from HR-HPV self-sampling since rates of adequate screening are as low as 25% in some studies [32].

Fewer studies have evaluated the acceptability of HR-HPV self-sampling among WLH. While three U.S.-based studies suggest a universal acceptance of HR-HPV self-sampling among WLH, instructions and kits were provided in an in-person, clinical setting [33,34,35]. While we recognize that several studies have recently implemented unsolicited, mail-based, and/or community health worker-guided HR-HPV self-testing in the U.S., none have explored facilitators or barriers to uptake in an HIV-positive population [14,17,36,37,38,39,40]. The current manuscript represents the first component of an exploratory mixed-methods study conducted to inform the development of the “*My* Self-Sampling, HPV Awareness, Results, and Empowerment” (*My*SHARE) intervention, which will examine the acceptability of HR-HPV self-testing among WLH. These preliminary data describe the knowledge, experiences, and needs of WLH regarding cervical cancer screening and seek to reconcile patient experiences with the views of their providers. Provider bias has been shown to have a significant impact on patients’ health care decision making. It is important to understand the screening needs among WLH to inform tailored interventions to maximize engagement, patient satisfaction [41], quality of life [16,17], and health outcomes [42].

## 2. Materials and Methods

### 2.1. Study Overview and Design

#### 2.1.1. Recruitment

Recruitment was performed using purposive sampling [43] for providers serving WLH and through convenience [44] and snowball sampling [45] for WLH. Organizations that provide care and services to WLH were solicited to distribute flyers to potential participants. ResearchMatch, a national health volunteer registry supported by the U.S. National Institutes of Health as part of the Clinical Translational Science Award program, was also used to recruit WLH. Participants were screened over the phone or online via Research Electronic Data Capture, a secure web-based application designed to support data capture for research studies and hosted by the Clinical and Translational Science Institute at Children’s National [46].

#### 2.1.2. In-Depth Interviews with Providers

We performed individual in-depth interviews (IDIs) with participants who had experience working with and/or providing health-related services to WLH (e.g., health care providers, HIV linkage-to-care specialists, community health workers, and leadership from partnering organizations). The semi-structured qualitative interviews used open-ended questions which allowed the providers to share a wider range of responses and allowed the research team to probe for a deeper understanding of the issues [47]. The interview questions, based on the Health Belief Model [48], covered three broad categories: (1) facilitators and barriers to cervical cancer screening uptake among WLH; (2) recruitment and retention strategies to reach WLH who do not regularly visit women’s health clinics; and (3) acceptability, feasibility and perceived effectiveness of promoting self-sampling devices as a supplementary method to increase cervical cancer screening uptake. Interviews were conducted either in-person in a private setting, or remotely via WebEx, and lasted no longer than one hour with the informed consent procedure to minimize participant burden.

#### 2.1.3. Focus Group Discussions with WLH

Participants were eligible for the focus group discussions (FGDs) if they (1) were a WLH 21 years old or older, (2) resided in the Washington-Baltimore Metropolitan Area (WBMA: DC, MD, and VA), and (3) were not currently participating in any other studies focusing on HR-HPV self-sampling for cervical cancer prevention. Discussions, lasting approximately one hour each, were held remotely via a pre-scheduled end-to-end encrypted WebEx meeting and facilitated by trained moderators and research staff. Once participants joined the call and privately identified themselves (prior to “entering” the group meeting and before the recording of the discussion was initiated), each participant was given a participant number and reminded to state their participant number before contributing to the discussion. Participants only used the phone feature, which increased confidentiality of participants, while the research team used the online WebEx application. The meeting was also locked once all expected participants had joined the call.

From the WLH’s perspective, topics discussed included but were not limited to: (1) cervical cancer prevention knowledge and screening behavior; (2) facilitators and barriers to cervical cancer screening uptake (e.g., insurance and income limitations; relationship with doctors and other health care providers); and (3) acceptability, feasibility, and perceived effectiveness of self-sampling devices (e.g., awareness of HR-HPV self-sampling, preference for self-/mailed- vs. in-office/clinician-collected sampling, concerns regarding self-sampling, recruitment and retention strategies to reach WLH in future trials). Upon completion of the FGDs, all participants were mailed a packet with information on HR-HPV, HR-HPR self-sampling, cervical cancer prevention, general health and wellness for WLH, and local and national resources for screening services.

### 2.2. Data Collection

Informed consent was obtained from the IDI and FGD participants. All discussions were digitally recorded, with participants’ approval. Gift card incentives of $35 and $25 were distributed to the providers and FGD participants, respectively, as a token of appreciation for their time and participation in this study.

### 2.3. Data Analysis

Unlike quantitative analysis which requires large samples, the goal of this study was to achieve thematic saturation which occurs when the collection of new data offers no additional insight [49]. All interviews and discussion sessions were transcribed verbatim for further review and analysis, coded, and subjected to thematic analysis. An inductive process, employing an open coding method, was adopted to examine the data from the IDIs and FGDs.

Two researchers experienced in qualitative research methods served as the lead for data analysis of the interviews (DL and AC). They independently reviewed four of the transcripts, then met to (1) discuss and develop a preliminary list of themes (codes), (2) define each theme (including determination of the inclusion and exclusion criteria for each), and (3) identify representative passages. Two additional coders were trained in the codebook using two to three interviews, and then codes were applied independently. Each transcript was thematically coded, using QSR International’s NVivo software (Version 12 Plus), by a minimum of two researchers and crossed-checked for agreement about the application of the codes. A taxonomy of emergent themes was developed and shared between all reviewers during data analysis as an iterative and collaborative process. Themes were organized into overarching domains and compiled with representative quotations. Discrepancies were resolved through discussion in a process of constant comparison.

### 2.4. Community-Engaged Approach

Community-engaged research requires partnership development, collaboration, and negotiation, as well as the commitment from both the community and academic researchers to address local health issues [50]. Community-engagement activities involved in this study included the following: (1) conducting formative research for intervention development; (2) setting this study in the community, at an agreed-upon location and time of convenience to the study participants; (3) securing buy-in and recruitment/retention support from local clinics and organizations that serve WLH; (4) forming a preliminary community advisory board; and (5) building in and carrying out member checks throughout this study.

### 2.5. Ethical Approval

All human participants were treated in accord with the Principles of the Ethical Practice of Public Health of APHA and gave their informed consent to participate. Review and approval for this study and all procedures was obtained from the George Washington University Institutional Review Board (Protocol NCR191689).

## 3. Results

IDIs with 10 providers and 6 FGDs with 39 eligible WLH, were convened between December 2019 and June 2020. The IDIs lasted an average of 38 min (range: 17–55) and were conducted with three nurse practitioners, three physicians, a social worker, a community health educator, a community advisory board member, and a clinical research manager. The majority (74.4%) of the FGD participants (WLH) were over the age of 51 and resided in Washington, DC (87.2%)—specifically, over half of the sample represented Wards 7 and 8 (56.2%), where the highest concentration of minority populations and lowest-grossing median household incomes in the District can be found. Twenty-five percent of the women in the FGDs were not eligible for routine cervical cancer screening due to self-reported history of hysterectomy or cervical cancer (Table A1 in Appendix A). While almost all women had been screened for cervical cancer in their lifetime (94.4%), only 77.7% indicated that they have had a Pap test within the last year (Table A1).

Major themes relevant to this paper were organized into five domains: (1) attitudes towards cervical cancer, (2) awareness and knowledge of cervical cancer and HR-HPV, (3) facilitators and barriers to screening, (4) awareness and knowledge of HR-HPV self-sampling, and (5) feasibility and acceptability of HR-HPV self-sampling. Domains and themes are presented with major representative key quotations from the individual and group interviews in Table A2 (Table A2 in Appendix A). Pseudonyms were assigned to the WLH and their age was included to provide additional contextual information. Inter-rater reliability was calculated for each code using the Kappa coefficient and found to be high (0.82–0.99) (Table A2).

### 3.1. Attitudes towards Cervical Cancer (from the FGD Participants’ Perspective Only)

Almost all FGD participants shared negative feelings associated with cervical cancer. They expressed their initial thoughts with terms such as “germs” “pain” and “fear”. While a few participants did not see cervical cancer as a present concern due to other priorities in their lives, most agreed that cervical cancer would be a significant life-threatening health event. Several women expressed hopelessness (“When I think about cervical cancer, I think about emptiness. Feeling empty because that’s […] one of the heartbeats that makes you a female.” Yasmine, 44) and fatalistic attitudes (“Cervical cancer is just something women die from.” Faith, 43), firmly believing that cervical cancer ultimately results in “death” (Lucy, 54).

Many of the women also spoke of their general concerns for, and susceptibility to, cervical cancer (Table A2). This was conveyed through their mentions of the importance of “detect[ing] cancer” (Joanna, 37), trying to “avoid getting cervical cancer” (Octavia, 54), and experiencing fear due to their lack of knowledge about cervical cancer. While some prioritized cervical cancer prevention and screening due to their high-risk, HIV positive status, or a family history of cancer, others indicated that they only prioritized cervical cancer screening after they encountered someone with cervical cancer and/or personally experienced an abnormal Pap or cervical dysplasia (“My sister died from [cervical cancer], so I get checked up for it. I had high-grade lesions and so my cervix was removed.” Grace, 63).

Participants further raised concerns about the added burden that a cervical cancer diagnosis would bring to WLH (Yasmine, 44), not only because of the associated physical pain and emotional baggage but also because of the need for intensive treatment and long-term follow-up care in the setting of other comorbid conditions. Some women additionally shared that they equated cervical cancer with no longer being able to bear children and raised concerns about the compounding psychological effects that infertility may bring to WLH (Yasmine, 44).

### 3.2. Awareness and Knowledge of Cervical Cancer and HR-HPV Infection

FGD participants acknowledged their own awareness and knowledge gaps, as well as their misconceptions, about cervical cancer and HR-HPV infection. For example, one participant shared, “I haven’t really heard a lot about HPV at all lately… to the point where I thought maybe [HPV] was absolutely cured or something.” (Melanie, 59). Others shared a desire to expand their knowledge of cervical cancer and HR-HPV: “I don’t know too much information about cervical cancer, but I really would like to know more about it.” (Victoria, 50). While many of the participants were able to identify that cervical cancer is cancer related to their reproductive organs (“You have cancer in the, I guess, the vagina area.” Lina, 66), only a handful could correctly distinguish what a cervix was and where it was located. There was also little to no knowledge of HR-HPV transmission or the role of HR-HPV in the development of cervical cancer. A few mentioned discussing cervical cancer and HR-HPV with their health care provider (“I heard about it from my doctor.” Holly, 56).

Among women who claimed some awareness and knowledge of HR-HPV, many credited the educational brochures and pamphlets made available in the waiting room of their doctors’ offices and advertisements and commercials about the HPV vaccine (Gardasil): “I haven’t seen that much about HPV. Sporadically I would see commercials but I think that, in my remembrance, they were geared more towards teenagers.” (Emma, 52). Most were aware that HR-HPV is a sexually-transmitted infection to which WLH are more susceptible (“Actually, my doctor told me that people with HIV are more prone to have it, HPV.” Iris, 59).

The women unanimously expressed the need to expand their current understanding of cervical cancer and HR-HPV. They identified peer group discussion, media, community outreach events, and providers as potential avenues to increase this dialogue among the community of WLH:

“We have our groups and stuff. We take notes down and they give us flyers, different information […] to keep us [knowledgeable]. I always keep my flyers and read over them.”

(Ivy, 61)

“I usually go to health fairs and I get information that way.”

(Lina, 66)

“I’ve never even seen anything, [not even] like that pamphlet in my doctor’s office, you know about HPV. Like I said, this is the first time I’ve ever heard of it… [There is a need for] educating physicians on educating their patients, even if they are not there [at their appointment] for that… outside of what we’re there for, to speak to them as far as HPV.”

(Dianne, 55)

Despite their low-level understanding of cervical cancer and HR-HPV, most WLH placed a high priority on cervical cancer screening: “I was going to say [that], to me, it’s a priority because I am HIV positive. […] It is still easier to get some infection even if I’m taking my medicine as usual so it’s a priority [for me] to get a Pap smear test every time required.” (Hope, 54).

The providers’ perceptions of the women’s awareness and knowledge of cervical cancer and HR-HPV accurately reflected that shared by WLH in the FGDs. The providers recognized that knowledge about cervical cancer among WLH could be improved and that it is vital for WLH to also understand the association between cervical cancer and HR-HPV (Community Advisory Member). Providers correctly believed that unless women had a history of cervical cancer or HR-HPV, their awareness and knowledge on these topics remained generally limited. However, providers underestimated the priority level of cervical cancer screening and prevention among WLH, assuming they would have many competing priorities (Social Worker).

### 3.3. Facilitators and Barriers to Screening

#### 3.3.1. Knowledge of Screening Guidelines

When asked about current cervical cancer screening recommendations for WLH (i.e., at HIV diagnosis but not before age 21) (Panel on Opportunistic Infections in Adults and Adolescents with HIV, 2021), the women provided mixed responses. While some accurately indicated that screening should be completed annually (or every 3 years if HR-HPV/Pap co-testing is performed or if the results of three consecutive Pap tests are normal) indefinitely, others believed that it should be completed twice a year and/or only up to the age of 65 years. Despite current guidelines/expert opinion to initiate screening no earlier than age 21, many women thought screening should be initiated on coitarche (“When you’re probably 18, definitely when you’re sexually active, that is when you definitely wanna get a Pap smear.” Fiona, 35), with a handful indicated that screening should be completed as early as the onset of menarche (Kylie, 60).

Interestingly, while providers universally agreed that cervical cancer screening for WLH is within the scope of primary care providers, they displayed varying knowledge of the screening standards specific to this patient population. While a few providers accurately recognized that (1) WLH should have a Pap test at the time of initial HIV diagnosis, then every 12 months (or every 3 years if the results of three consecutive Pap tests are normal) and (2) that screening (either cervical cytology only, or cytology and HR-HPV co-testing for WLH ≥ 30 years) should continue through a WLH’s lifetime (Panel on Opportunistic Infections in Adults and Adolescents with HIV, 2021) (“A lot of the patients we see are older. The guidelines tell [us] that after age 65, you don’t have to do it anymore in the general population, but the guidelines we follow here is once a year [for WLH].” Physician), others were not aware of specific screening recommendations for WLH:

“So I don’t think the guidelines are any different for women with HIV. Generally, it used to be every three years [that] this has to be done and now it’s every five years, unless, of course, they have something abnormal. […] Oh okay, is that the current recommendation for those with HIV? I didn’t even know that.”

(Nurse Practitioner)

#### 3.3.2. Screening Behavior

Most participants indicated that they had regular cervical cancer screening (Natalie, 54). However, a handful of women explicitly stated that they only completed cervical cancer screening tests when they had symptoms or if it was directly recommended by their provider (Julia, 50). Some of the women similarly voiced that if cervical cancer screening was not brought up by their physicians, they would not request to be screened (“Oh no, no, I would never request that.” Joanna, 37).

WLH felt that providing easily accessible general education on cervical cancer and HR-HPV prevention would increase screening intention and behavior (“Some people just don’t know and you have to educate them. […] It’s something that’s helpful to save your life.” Natalie, 54). They felt that education targeting all ages of WLH and emphasizing their increased risk of cervical cancer and HR-HPV would be welcome and effective.

Providers also recognized the importance of physician recommendation for screening and agreed that WLH are generally open to screenings and compliant with regular cervical cancer screening (“I see a lot of women who are HIV positive and, in general, [for their] annual screening.” Physician). They further shared their perception that older WLH were less interested in regular screenings compared to younger WLH (Physician). Like WLH, providers emphasized the importance of education for young WLH (Dianne, 55):

“Right now, our team is having a hard time engaging the young adults living with HIV in care for that [cervical cancer screening]. […] Once we get them in the office or [on a] phone call, we can talk to them more about the risks for cervical cancer and why it’s important for them to do the screening.”

(Nurse Practitioner)

### 3.4. Awareness and Knowledge of HR-HPV Self-Sampling

While WLH had little awareness or knowledge of HR-HPV self-sampling, some inferred that self-sampling would be similar to cervical cytology (Bethany, 58). Others supposed it would be similar to a urine test (“I would think that it would be maybe like a first morning urine, it’s like you’re doing a clean catch urine. Like the first urine you do in the morning to catch the specimen when you don’t have no water in your body. I don’t know, that what I would think it would be.” Wendy, 55). Participants who had previously completed an at-home self-test for sexually-transmitted diseases or infections (STDs/STIs) were more likely to accurately describe the HR-HPV self-sampling process:

“I would think that they would maybe send a Q-tip or something and put it inside of a tube […] and you send it back. […] I think I had something done like that before, […] I think I had like an anal test done.”

(Ivy, 61)

Most providers had a similar lack of awareness or knowledge of HR-HPV self-sampling and equated it to other STD/STI self-tests:

“In my mind, it would be something pretty simple and not very invasive; not time-consuming. I mean, almost like swabbing in your cheek or in your mouth for other things, but this time, you’re swabbing the vaginal area.”

(Community Advisory Member)

### 3.5. Feasibility and Acceptability of HR-HPV Self-Sampling

WLH felt HR-HPV self-sampling was feasible and acceptable due to its convenience (e.g., physical accessibility; Kayla, 65), privacy, simplicity, and ease of use (“Because it’s simple and not difficult, I can do it.” Giselle, 53). Many were motivated by the health benefits of HR-HPV testing (“Motivation for wanting to take that test would be to make sure everything is in line, that we’re healthy. You know, ensuring health.” Fiona, 35) and some elaborated that knowing how to correctly do the test and being empowered to take care of their bodies would serve as additional facilitators (“Yeah, I want to know what’s going on with my body” Coralie, 61; Natalie, 54). Affordability and provider recommendation were also mentioned as important factors (“If it was free, I might do it but if it has a cost, [that] might deter people.” Grace, 63; Ursa, 46). Although the majority of our FGD participants expressed willingness to try HR-HPV self-sampling (80.5%), a few shared their preference for in-person screening with their provider (“I prefer coming into the clinic and letting them do it for me […], just to be sure [that] it’s done accurate and done right.” Wendy, 55).

Most providers agreed that HR-HPV self-sampling was not frequently offered to WLH (“There may be a few providers who do it, but it’s not widely used currently or recommended.” Physician). Some providers recognized the use of HR-HPV self-sampling for patients with gender dysphoria or a history of sexual abuse:

“I know that some providers offer the test, [but] I don’t know exactly how they’re doing it. […] [Some providers are] giving sampling kits to patients with gender dysphoria who don’t want to do the Pap; or it’s too like traumatic to do it so they’ll give [their patients] something that they can take home to do or that they can do in the clinic on their own.”

(Physician)

Providers had mixed opinions on whether HR-HPV self-sampling should be offered in the U.S. and whether acceptability and uptake among the WLH would be high. Most providers were concerned about incorrect use of the kit and the capacity for follow up and triage to further testing or treatment in the case of an abnormal test (“It’s one thing to do the screening but then it’s like access to follow-up. Because if it’s HPV positive, do we have the providers who are doing the colposcopy and things like that?” Physician). Overall, providers believed that HR-HPV self-sampling would be feasible for WLH. The providers highlighted the “convenience” and “privacy” that self-sampling would offer its users (Nurse Practitioner).

#### Remote HR-HPV Self-Sampling

In our study, WLH were overwhelmingly willing to try remote self-sampling, emphasizing “one less trip to the doctor’s” (Kayla, 65). Many of them felt self-sampling was especially crucial in situations such as the COVID-19 pandemic where patients do not feel in-person visits are safe (“Um, going into the office right now for a Pap smear, and stuff like that, is very dangerous so we really need one you can do at home.” (Coralie, 61). The women strongly favored a mail-based intervention (vs. having to physically pick up the self-collection kits from their provider’s office or a nearby pharmacy), highlighting its convenience and privacy:

“I think mailing it out to them works best because [after] receiv[ing] it, [they can] just do it in the privacy of their own home, then mail it back in. Just [include] a self-addressed envelope; [they can] put it in there and no one [will] know what is in that envelope [when they] mail it back.”.

(Holly, 56)

“Because [they] have kids and [are not] able to get out, mailing them [an HPV self-collection kit] would be one way for them to get it and [to] get [screening] done.”

(Page, 62)

Many providers were concerned that remote self-sampling and the self-sampling process would be an added burden for both providers and patients (“It feels maybe a little burdensome… [It would be] a little bit challenging to ask people to do something at home and then bring it back in. That’s like two things for them to do.” Nurse Practitioner). They feared mailed kits would not be returned promptly or at all. These concerns, however, were contradictory to what the majority of our FGD participants shared.

## 4. Discussion

This qualitative study describes the experiences and knowledge of cervical cancer and HR-HPV among WLH in the greater Washington-Baltimore area and examines the feasibility and acceptability of HR-HPV self-sampling for this cohort. To our knowledge, this is the first qualitative study to examine these questions among a diverse, urban population of WLH and their providers. Since providers of health care and social services directly influence their patients’/clients’ decision making, health-seeking behaviors, and access, their perspectives will undoubtedly influence the acceptability and feasibility of HR-HPV self-sampling.

The present study found that, overall, WLH have negative (e.g., fearful and anxious) attitudes towards cervical cancer and acknowledge the severity of the disease. Both providers and WLH expressed that cervical cancer and HR-HPV knowledge among WLH is limited. Patients and providers agreed that this knowledge should be improved through patient education to help women prioritize cervical cancer screening despite competing needs. Our study revealed provider recommendation as a major driver of cervical cancer screening. While our cohort had little or no prior knowledge or experience with HR-HPV self-sampling, they were very accepting of such testing. Providers were also unfamiliar with HR-HPV self-sampling and recognized its appeal to patients, despite the administrative and clinical challenges for follow-up of abnormal results.

Similar to prior literature, our study revealed knowledge gaps regarding HR-HPV and cervical cancer among WLH [14,15,16]. FDG participants and providers recognized the importance of educational materials explaining the etiological role of HR-HPV infection in cervical cancer, current screening guidelines, impact of a comorbid diagnosis, long-term side effects (e.g., infertility), and benefits of regular screening. They endorsed that an emphasis on the aforementioned content areas would be most effective, as revealed in prior studies [14,15,16].

Our study highlights the importance of provider recommendation in cervical cancer screening, with mixed responses to provider-led decision making. Although FGD participants ultimately felt responsible for their own health, they deferred to their providers’ recommendations and felt they would be less likely to bring up cervical cancer screening unprompted. Similar findings were reported by Beach et al. [51]: 86% of participants preferred shared decision making or for their physician to make all the decisions [51].

Our study is the first to explore the acceptability of HR-HPV self-sampling from both patient and provider perspectives. While some of the providers believed that HR-HPV self-sampling would be burdensome, our FGD participants were extremely receptive to the concept of remote self-sampling and believed they would complete this testing if the self-collection process were simple with clear instructions. Most women, in our study, were familiar with other self-testing options (e.g., for colorectal cancer or other STIs) which may further explain their receptiveness to HR-HPV self-sampling. Findings from our study also distinctly emphasized the need for designing interventions that offer remote HR-HPV self-sampling, especially to women with limited access to cervical cancer screening, so that they can complete self-collection at a time and place that is convenient and appropriate to them.

### 4.1. Limitations

There are several limitations of the current study. First, the findings described herein reflect primarily the views and experiences of WLH residing in the urban WBMA and may not be generalizable to that of WLH located in more rural areas. Additionally, since most women in our FGDs were over the age of 50, our findings may not be generalizable to a younger population. Lastly, we conducted our FGDs without video, in order include those without camera-enabled devices or internet connection. Therefore, visual cues were not available to the discussion moderator. Despite these limitations, this study adds important information to the literature related to cervical cancer prevention efforts among vulnerable populations and provides valuable insights that can help shape policies and programs aimed at addressing the health needs of WLH, as defined by the women and the providers that serve their community.

### 4.2. Implications for Practice and/or Policy

As outlined in the Healthy People 2030 objectives, increasing the number of women who are screened based on the most recent guidelines [52] will reduce cervical cancer mortality [53]. As WLH are at substantially increased risk for HR-HPV infection and cervical cancer due to their immune-compromised status, identifying effective cervical cancer prevention strategies and screening services may help reduce premature mortality in this group. The experiences qualitatively identified in this study may be used to guide patient-centered communication aimed at improving awareness and knowledge of HR-HPV infection prevention among WLH and to inform interventions designed to increase cervical cancer screening among this vulnerable population. One such intervention, as endorsed by the WLH in this study, involves the opportunity to remotely complete an HR-HPV self-sampling test kit. Our next step is to implement and evaluate the feasibility and preliminary efficacy of a mail-based HR-HPV self-sampling intervention among WLH, while identifying barriers and intervention preferences. This will ultimately inform the development of a larger-scale study that will incorporate health information explicitly tailored to this priority population.

## 5. Conclusions

WLH are a particularly vulnerable population that faces several barriers to cervical cancer screening [9,54,55]. HR-HPV self-sampling is appealing to our study population and could reach these under-screened women by bypassing the typical barriers to office-based screening. Physician recommendation for HR-HPV testing was an important factor in patient decision making, highlighting the importance of physician recommendations, even for at-home self-sampling. Our qualitative study identified multiple barriers to HR-HPV testing that can be overcome by self-collection. Developing and disseminating acceptable, efficient, and cost-effective screening options are paramount to eliminating cervical cancer disparities.

## Data Availability

Daisy Le had full access to all the data in this study and takes responsibility for the integrity of the data and the accuracy of the data analysis.

## Appendix A

**Table A1:** Characteristics of women living with HIV who participated in the focus group discussion (n = 39).

Characteristics of FGD Participants	Number (Percent)
Age, years	
31–40	3 (7.7%)
41–50	7 (17.9%)
51–60	20 (51.3%)
61–70	9 (23.1%)
Median (range)	55 (35–66%)
Residence	
DC	34 (87.2%)
MD	3 (7.7%)
VA	2 (5.1%)
History of cervical cancer or hysterectomy	
Yes	10 (25.6%)
No	29 (74.4%)
Residing in the District of Columbia * (N = 32)	
Ward 1	4 (12.5%)
Ward 2	2 (6.3%)
Ward 4	1 (3.1%)
Ward 5	3 (9.4%)
Ward 6	4 (12.5%)
Ward 7	5 (15.6%)
Ward 8	13 (40.6%)
Residing in Maryland (N = 3)	
Baltimore County	1 (33.3%)
Prince George’s County	2 (66.7%)
Residing in Virginia (N = 1)	
Richmond County	1 (100%)
Ever had a Pap test (N = 36)	
Yes	34 (94.4%)
No	1 (2.8%)
Don’t know	1 (2.8%)
Had a Pap test in the past 12 months (N = 36)	
Yes	28 (77.7%)
No	6 (16.7%)
Don’t know	1 (2.8%)
Missing	1 (2.8%)
Willing to collect own sample for cervical cancer screening (N = 36)	
Definitely not willing	2 (5.6%)
Probably not willing	2 (5.6%)
Not sure	3 (8.3%)
Probably willing	9 (25.0%)
Definitely willing	20 (55.5%)

* For municipal purposes, including local elections and city planning, Washington, D.C. is divided into eight wards—each represented by its own councilmember.

**Table A2:** Domain and themes from providers and women living with HIV.

Domain	Themes	WLH	Providers
2.1. Attitudes towards cervical cancer			
2.1.1	Fear	“[…] I mean, it’s just scary to me when I think about it and I’m over 50 now. It’s real scary.” *(Alissa*, *59)*	
2.1.2	Sickness and pain	“I think about pain and that.” *(Wendy*, *55)*	
2.1.3	Childbearing concerns	“You know, what comes to mind with cervical cancer, I think of, definitely not being able to have children and just a cancer that, in your reproductive areas.” *(Yasmine*, *44)*	
2.1.4	Burdensome treatment	“A constant, being constantly being picked and probed at, spotlight, spotlight. Negatively.” *(Yasmine*, *44)*	
2.1.5	Cervical cancer as a priority	“Well, because half of family has cancer in them. So, I’m not trying to get it, you know. They smoke a lot of cigarettes and all of that. They’ve never had cancer but most of them die from everything besides that, so I’m trying to keep being healthy, right. I’m HIV positive so I’m trying to keep myself healthy and everything […].” *(Octavia*, *54)*	
2.1.6	Cervical cancer not a priority or concern	“No, it wasn’t something that I would think about or be on the top of my list because that’s something, it’s just not something to really, that I would focus on, I’ll put it that way.” *(Giselle*, *53)*	“I really think raising awareness about cervical cancer, um, because there are, I just don’t think that there are enough conversations about it for people to even think or even know that it’s a concern.” *(Social Worker)*
2.2. Awareness and knowledge of cervical cancer and HPV			
2.2.1	Limited awareness and knowledge	“I don’t know anything much. I really don’t know anything much about it. I’ve heard advertisements about young, younger adults getting tested or teenagers getting tested to prevent HPV but that’s about all I knew of it.” *(Emma*, *52)*	“I think the knowledge generally is, is very low. I think it may become, women become aware only when they are confronted with, with the diagnosis. But I think before then, you, certainly I never hear people, women talking about it, you know, or, I’ve just never heard conversations and I’ve been around women, women’s organizations, women clients, women with HIV, and women who also, they have concerns about cancer, but they, I’ve never heard in my circle of people that I come in contact with any conversations about it. So I think it’s something that is not known very much.” *(Community Advisory Member)*
2.2.2	Knowledgeable of their risks	“With HIV, I believe that we are more prone to infections so there is more chance of getting the cervical cancer.” *(Hope*, *54)*	“…but they are not aware of their increased risk for HPV and cervical cancer.” *(Nurse Practitioner*)
2.2.3	Early detection as prevention	“They’ve got, other cancers that are detected earlier, they’ve got a bigger survival rate. I just think that earlier detection is the best.” *(Ursa*, *46)*	
2.2.4	HPV vaccination as prevention	“I think we should get vaccinated for HPV and we should practice safe sex, if I can say that.” *(Faith*, *43)*	“So they, I feel like they, we make women living with HIV more aware that of that necessity, but I have had that instances where I had to explain to them, the purpose of the HPV vaccine.” *(Nurse Practitioner)*
2.2.5	Cervical cancer and HPV not preventable	“I don’t think there is anything you can do to prevent it.” *(Joanna*, *37)*	
2.3. Facilitators and barriers to screening			
2.3.1	Understanding of screening guidelines	“Yeah, because when you start your menstrual cycle your body changes. So, you need to be aware of a lot of things.” *(Kylie*, *60)*	“I think they know that routine screenings are required. And I think that their main primary care provider let them know that the guidelines are, are different for women living with HIV then they are for the general population.” *(Physician)*
2.3.2	Screening behavior	“I do it annually but if something is going on, I’m gonna call the doctor and go see what’s going on.” *(Natalie*, *54)*	“…aside from, I mean obviously we know that they are generally uncomfortable, but like I haven’t specifically had people be like, I’m not getting that done, you know, because it hurts or something like that.” *(Nurse Practitioner)*
2.3.3	Sub-population screening habits	The thing is, it’s like a lot of our youth these days, they’re not even considering, they don’t pay attention to their health. A lot of youth these days, the only time they would visit a doctor maybe, is if it’s an emergency, you know like, they are hurt real bad, emergency room because their stomach is hurting really bad, in pain. You know, a lot of youth, it’s not like a lot of people coming up. We were more responsible. We knew when to go to the doctor and everything. It’s not like that now. But, it will be nice if that could happen with the youth.” (*Dianne*, *55*)	“So I think for most women in the general population, and with HIV, um, screening exam becomes harder to stomach as you get older and you don’t use vagina or think of your vagina less and less on daily basis you know what I mean. When you’re either engaging in frequent sexual activity or still childbearing or stopped menstruation, post-menopausal women, I think they’re more reluctant to get a pelvic exam that include the pap and that kind of invasive procedure.” *(Physician)*
2.3.4	Screening recommendation	“I get it when my doctor recommends.” *(Julia*, *50)*	“I think it boils down to how, I mean are they being followed regularly by a provider, if so, then I think it will be helpful for that provider to spend a few minutes during the appointment to talk about screening and just to briefly touch on it to give them a reminder. But the way how, like I said, the way how it’s done here, the, their primary provider is usually looking through their labs to see if their up to date.” *(Physician)*
2.4. Awareness and knowledge of HR-HPV self-sampling			
2.4.1	Limited awareness and knowledge	“Oh, no, I haven’t heard about the test until now.” *(Grace*, *63)*	“No. Nobody I ever practiced on, has had any question about that. So I don’t know if they know about it or not. No one has ever brought it up to me. And I actually never heard of it until the study design.” *(Physician)*
2.4.2	Knowledgeable about HR-HPV self-sampling	“I think it’s just pretty much like a Pap smear isn’t it, pretty much, and taking cells and viewing what the cells hold or have in them.” *(Bethany*, *58)*	“Yeah, I mean, I do think HPV self-sampling is feasible, it’s been shown to be accurate in several large studies, compared, especially compared to like Pap smear alone, HPV self-sampling, HPV is more, sensitive for cervical cancer screening than Pap smear. So it should be feasible.” *(Physician)*
2.4.3	Comparison to other self-sampling tests	“Okay, the test is, it’s not that hard. Some of them they give you a cream to put on the like the q-tip, just like you put a tampon in, it’s like you insert this thing. There’s a tube to send it back to them and then they’ll tell you whether you have cervical cancer or not once they’ve screened it. It’s not hard to do, it’s just like, it’s just an easy Pap smear. You’re doing it at home by yourself.” *(Holly*, *56)*	“I kind of think of you, kind of, like kind of getting, this is going to sound weird, but kind of getting weird of like for a colon cancer screening, like you just do it at home and then send it off, but you don’t have to take off work or get to the doctor, you just do it whenever you can, send it off whenever you can, and wait for the results.” *(Community Advisory Member)*
2.5. Feasibility and acceptability of HR-HPV self-sampling			
2.5.1	Convenience as a motivating factor	“I would want to use it, just one less trip to the doctor’s.” *(Kayla*, *65)*	“I think definitely. I think just having that convenient option, especially when they are like rushing to get out of the appointments to go somewhere else and being like here, take the kit and mail it, it’s free to mail, just drop it off or you can do it really quickly and leave it, and that privacy I feel like is very convenient.” *(Nurse Practitioner)*
2.5.2	Privacy as a motivating factor	“[…] I would do it because sometimes, like when I go to the doctor and I have a Pap smear if I smell uncomfortable or feel uncomfortable in that area, I feel embarrassed.” *(Ursa*, *46)*	“I think just like that privacy, is a good facilitator for that use.” *(Nurse Practitioner)*
2.5.3	Provider recommendation as a motivating factor	“I would, I mean if I, put it like this, say if I went to the doctor and he offered me, he started telling me about a home kit then I probably would try it at home.” *(Ursa*, *46)*	So I think when you have an atmosphere where, where you have those kinds of relationships with your provider, that are respectful and trusting and whatnot that I think, I think you would be very open to hearing about the opportunity to test yourself. *(Community Advisory Member)*
2.5.4	Self-efficacy and education as motivating factor	“More knowledge about it before actually sending out a test. I wouldn’t say waste the money on sending out a test if it’s not gonna be used or people not knowing what it’s used for.” *(Emma*, *52)*	
2.5.5	Empowerment as motivating factor	“Well, I think what will probably be empowering is you knowing what’s going on with your body, as well as taking the test and seeing the results, well you know, what the result and the outcome will be. If anything, if something is going on, early detection.” *(Natalie*, *54)*	The convenience and, and some power around, being able to, to test yourself. *(Community Advisory Member)*
2.5.6	Promising uptake by WLH	“I wouldn’t mind, um, doing it, cause it gives me a sense of taking control of my health.” *(Yasmine*, *44)*	“Yeah, I mean, right now we do, we do self-tests for vaginitis and I think people prefer it.” *(Physician)*
2.5.7	WLH unlikely to use HR-HPV self-sampling	“No, I’d rather want a doctor to do it.” *(Lucy*, *54)*	“Are we really going to get this back?” So that feels maybe a little bit challenging to ask people to do something at home and then bring it back in. That’s like two things for them to do.” *(Nurse Practitioner)*
2.5.8	Remote (at-home) HR-HPV self-sampling	“Okay, that was one of the questions, the one, but I’ve never heard of it, but I think it’s, uh, really needed. You know, because times like now, now with […] the pandemic going on, you could do it at home like I was sick and I had straight to a care, I didn’t want to go up to a hospital because I know it was crowded and crowded, so, I think it’s really needed that you could do the one at home.” (*Coralie*, *61*)	
2.5.9	Mail-based	“Nowadays, I think it would be best to mail it because not too doctors are accepting people into their offices nowadays, you know, so it would be best to mail it.” (*Grace*, *63*)	
